# An annotated checklist of the Pyralidae of the region of Murcia (Spain) with new records, distribution and biological data (Lepidoptera, Pyraloidea, Pyralidae)

**DOI:** 10.3897/BDJ.10.e79255

**Published:** 2022-03-14

**Authors:** Manuel J. Garre, John Girdley, Juan J Guerrero, Rosa M. Rubio, Antonio S. Ortiz

**Affiliations:** 1 Universidad de Murcia, Murcia, Spain Universidad de Murcia Murcia Spain

**Keywords:** Lepidoptera, Pyralidae, checklist, chorology, distribution, new records, phenology, Iberian Peninsula

## Abstract

**Background:**

The Murcia Region (south-eastern Iberian Peninsula) has a great diversity of Lepidopteran fauna, as a zoogeographical crossroads and biodiversity hotspot with more than 850 butterflies and moth species recorded.

**New information:**

In the present paper, based on an examination of museum specimens, published records and new samples, a comprehensive and critical species list of Pyralidae moths (Lepidoptera, Pyraloidea) is synthesised. In total, three subfamilies, 67 genera and 142 species have been recorded and these are listed, along with their collection, literature references and biological data, including chorotype, voltinism and the flight period in the study area. The subfamilies are Galleriinae, Phycitinae and Pyralinae. Seventy-three species are newly recorded, sixty-two species are confirmed from literature and only seven species have not been observed for the Murcia Region.

## Introduction

The Pyralidae, belonging to the superfamily Pyraloidea, are mainly nocturnal micromoths (Microlepidoptera) with an estimated 6,000 named species worldwide, of which the European fauna is represented by ca. 470 species (Leraut 2014). In the Iberian Peninsula, 262 species have been recorded (Vives-Moreno 2014). The two main evolutionary lineages within Pyraloidea, Pyralidae and Crambidae, are monophyletically distinguished by the morphology of tympanal organs (Minet 1982, Minet 1983, Slamka 2006). Pyralidae are characterised by the forewing venation with R5 stalked or fused with R3+4 and without oval sclerotisation costad on the base of vein A; presence of paired tympanal organs situated ventrally in the second abdominal segment with tympanum and conjunctivum in the same plane; tympanal chamber cephalad closed and accessory tympana absent in metathorax; lobulus and praecinctorium are absent; male genitalia with uncus arms; and segment A8 of larvae almost always with sclerotised ring around base of seta SD1 (Goater et al. 2005, Slamka 2006).

The Pyralidae of Europe have been relatively well studied, although there is a need for further investigation on habitus and distribution. The Southern European and, especially, Iberian species are poorly recorded and more precise data are necessary for the production of distribution maps. Historically, the first pyralid moth recorded and described from the Murcia Region was *Hypotiamiegi* (Ragonot 1895) and, later on, *Hypotialeucographalis* (Hampson 1900) was also described. Caradja (1910) recorded *Acrobasiscentunculella* (Mann), *Acrobasisobliqua* (Zeller), *Amphithrixsublineatella* (Staudinger), *Epischniaillotella* Zeller, *Homoeosomanebulella* (Denis & Schiffermüller), *Homoeosomasinuella* (Fabricius) and *Pterothrixidiarufella* (Duponchel) and Zerny (1914) described *Aphomiamurciella* from Sierra Espuña and recorded *Alophiacombustella* (Herrich-Schäffer), *Asalebriaflorella* (Mann), *Assaraconicolella* (Constant), *Dioryctriasylvestrella* (Ratzeburg), *Ephestiawelseriella* (Zeller), *Euzopheralunulella* (O. Costa), *Psorosamediterranella* Amsel, *Aglossabrabanti* Ragonot, *Lorymaegregialis* (Herrich-Schaffer) and *Stemmatophoravulpecalis* Ragonot. Subsequently, Caradja (1916) confirmed *Aphomiamurciella* Zerny and recorded *Eurhodopecruentella* (Duponchel), *Stemmatophoragadesialis* Ragonot and *Synaphediffidalis* (Guenée). Schmidt (1934) described *Asalebriapseudoflorella* from Sierra Espuña, considered as a subspecies of *A.ferruginella* (Zerny) at the present time.

Later, Agenjo (1948) described, also from Sierra Espuña, *Epischniaperoni*, currently synonymised with *Epischniaasteris* (Staudinger) and *E.prodromella* (Hübner) and *E.illotella* (Zeller) were recorded for the first time, while Agenjo (1952) recorded *Cryptoblabesgnidiella* (Millière) and *Pempeliapalumbella* (Denis & Schiffermüller) amongst others, although *Coenochroaablutella* (Zeller) and *Homoeosomanimbella* (Duponchel) (cited as *Homoeosomasubalbatella* Mann) have not been collected during the present study. Subsequently, Amsel (1955) described, also from Sierra Espuña, *Archiephestiamurciella* considered as a synonym with *Archiephestiaadpiscinella* (Chrétien) and Agenjo (1962) recorded *Ancylosisuncinatella* (Ragonot) and *Hypotiamiegi* (Ragonot).

Subsequent contributions are those of Roesler (1973), Derra and Hacker (1982), De Prins (1985), Vives-Moreno (1992), Asselbergs (1993), who recorded *Merulempistaturturella* (Zeller) (cited as *M.numidella* (Ragonot)), Slamka (2006), Knölke (2007), Pérez de-Gregorio and Requena (2008a), Pérez de-Gregorio and Requena (2008b), Pérez de-Gregorio and Requena (2008c), Slamka (2010), Pérez de-Gregorio and Requena (2010), Palacios and Abad (2010), Palm (2012), Leraut (2014), Pérez de-Gregorio and Requena (2014), Vives-Moreno (2014), Slamka et al. (2018), Slamka (2019) and Bidzilya et al. (2020).

Recently, *Pseudoinsalebriaiberica* Slamka et al. 2018 and *Gymnancylahillneriella* Gastón and Vives 2018 have been described from Murcia and *Ceutolophaisidis* (Zeller) has been recorded by Girdley et al. 2018, *Gymnancylahornigii* (Lederer) by Girdley et al. 2019 and *Psorosaferrugatella* (Turati) and *Epischniaampliatella* (Heinemann) by Girdley et al. 2020.

The Region of Murcia has a great diversity of Lepidopteran fauna, as a zoogeographical crossroads and biodiversity hotspot, with more than 850 butterfly and moth species (Ortiz et al. 2016, unpublished data). The summary ecophysiological characterisation of the study area can be consulted in Garre et al. (2021).

**Figure 1.**
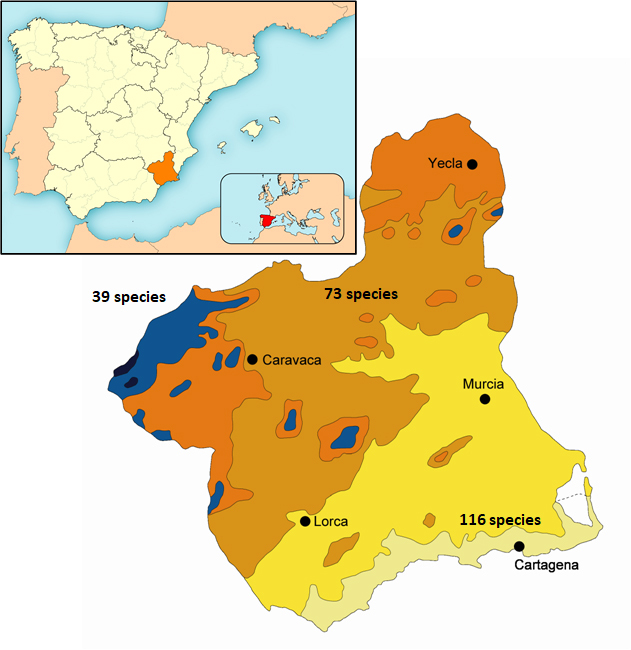
Map of the known species diversity in the bioclimatic areas in the Murcia Region. Black and blue: Oro- and Supramediteranean; orange and light brown: Cold and mild Mesomediterranean; Yelow and light green: Upper and lower Thermomediterranean.

Considering various bioclimatic approaches relative to temperature (thermotypes) and rainfall (ombrotypes), four different bioclimatic areas can be recognised according to Alcaraz et al. (2008): thermo-, meso-, supra- and oromediterranean (Fig. 1). Climatic and geological interactions differentiate a great variety of habitats as thermoxerophylic on the sunny slopes of the mountains and, on the other hand, as mesophylic in depressions or very dark exposures, in riparian zones amongst halophytic vegetation and on sandbanks and dunes from the inland to the coastal areas along with agricultural crops and anthropophilic areas. Altogether, they make up ten habitats and 47 special terrestrial conservation areas of community importance (Alcaraz et al. 2008).

This present checklist is intended to update the recorded species and to facilitate access to the most recent data on the Pyralidae family from the Murcia Region (south-eastern Iberian Peninsula) for taxonomists providing data about distribution, chorology, phenology and voltinism.

## Materials and methods

Adult specimens were examined externally and the genitalia structures were dissected using standard procedures (Leraut 2014) with minor modifications with the use of DMHF (2,5-Dimethyl-4-hydroxy-3(2*H*)-furanone). Roesler (1973), Slamka (2006), Leraut (2014) and Slamka (2019) were consulted mainly for identifications. Alpha diversity Simpson (Simpson 1949) and Chao1 (Chao 1984) indices, applied to abundance data on 136 species collected, were calculated in PAST software v. 4.0.9 (Hammer et al. 2001).

The list contains all species of Pyralidae collected by the authors until the end of 2021, along with the material deposited in the private collections of J.A. de la Calle, F. Lencina, F. Albert and F. Arcas. It also includes all of those records previously referenced in the bibliography.

Black and actinic (6 and 15 W) Heath traps, 125 W Robinson traps, 125 W mercury vapour traps and 4 W LED light traps were used for nocturnal sampling. Catches taken during daytime and in the urban environment (street lighting) are also included. All these sampling points are located within the study area and, especially, in the natural protected areas like the mountainous Parks of Sierra Espuña, Sierra de la Pila, El Valle and Carrascoy etc. and the coastal Parks of Calblanque, Monte de las Cenizas and Peña del Águila, Salinas and Arenales de San Pedro del Pinatar, etc.

### Notes on the checklist

The subfamilies are systematically ordered and identified, based on the most recent classification of Pyralidae by Nuss et al. (2021), Vives-Moreno (2014) and Slamka (2019) with minor modifications. The genera and species are listed under their subfamilies and are also ordered systematically, together with collection data (sampling localities, altitude, decimal coordinates, date and number of specimens). In addition, for each species, related references and biological data are provided, including general chorotypes and Iberian endemism, voltinism based on literature and the flight period in the study area or nearby areas indicated by months in Roman numerals. All studied specimens are deposited in the entomological collection in the Zoology Department of Murcia University (Spain) and in the collections of Francisco Lencina, Fernando Albert and Francisco Arcas. The occurrence data can be accessed at DOI:https://doi.org/10.15470/a6fcav

Roesler (1973), Slamka (2006), Leraut (2014) and Slamka (2019) were consulted to obtain the information on biology, voltinism and geographical distribution of the species, while Calle (1982) and Varga (2010) were consulted for biogeographic criteria. The voltinism of some species is unknown and data in text have been made, based on our observations in the study area.

## Checklists

### Annotated checklist of Pyralidae recorded in the Murcia Region

#### Pyralidae

B1365F49-907C-52FF-BAA3-F17166C467DF

#### Galleriinae

5AE84485-CB19-5C81-B70F-C6364D0CCDFA

#### Achroia grisella

(Fabricius, 1794)

0A6192CD-1A75-51D2-8019-C5557A4D4004

##### Distribution

Cosmopolitan

##### Notes

Biological data: Polyvoltine. Flight period: VI, X. First record in Murcia Region.

#### Galleria mellonella

(Linnaeus, 1758)

FAE03D7A-7152-5811-AA48-B745BA7C5155

##### Distribution

Cosmopolitan

##### Notes

Biological data: Polyvoltine. Flight period: VII-X. First record in Murcia Region.

#### Cathayia insularum

(Speidel & Schmitz, 1991)

4B46AEEC-5E64-54CC-8086-65B991E76888

##### Distribution

Atlanto-Mediterranean

##### Notes

Biological data: Polyvoltine. Flight period: I, VII-IX. First record in Murcia Region.

#### Aphomia sociella

(Linnaeus, 1758)

35FEB8CA-0719-5C9E-AD36-4447AB07C1F4

##### Distribution

Holarctic

##### Notes

References: Pérez de-Gregorio and Requena (2010). Biological data: Univoltine. Flight period: V-VII.

#### Aphomia murciella

Zerny, 1914

F478453F-F3FF-594E-AF22-D763E6F797B9

##### Distribution

Endemic

##### Notes

References: Zerny (1914), Caradja (1916), Slamka (2006). Biological data: Univoltine. Flight period: VII-VIII.

#### Aphomia sabella

(Hampson, 1901)

A0ECD27D-0594-517C-8C0D-BC2F7BCB6C26

##### Distribution

Mediterranean-Asiatic

##### Notes

Biological data: Polyvoltine. Flight period: VII-VIII. First record in Murcia Region.

#### Aphomia zelleri

(Joannis, 1932)

F0FBDD9A-7EB6-524E-A063-D49BAC5FD1E4

##### Distribution

Eurasiatic

##### Notes

References: Palacios and Abad (2010). Biological data: Univoltine. Flight period: IX-X.

#### Lamoria anella

(Denis & Schiffermüller, 1775)

2272FDA4-F131-5F10-8849-4F557F45B70F

##### Distribution

Cosmopolitan

##### Notes

Biological data: Bivoltine. Flight period: IV-XI. First record in Murcia Region.

#### Phycitinae

17B0D194-E067-5F05-B5E7-6D810A60FFC4

#### Coenochroa ablutella

(Zeller, 1839)

0E2AD0D4-FCB2-5ADE-AC67-4C414D6ADA8D

##### Distribution

Tropical

##### Notes

References: Agenjo (1952). Biological data: Bivoltine. Flight period: IV-X.

#### Peoria cremoricosta

(Ragonot, 1895)

7DB78095-D32B-5AFE-B9DA-0C6EFE0C89CA

##### Distribution

Mediterranean-Asiatic

##### Notes

Biological data: Bivoltine. Flight period: IX. First record in Murcia Region.

#### Peoria translucidella

(Chrétien, 1911)

789DBD4B-BF8A-56C0-931D-B039BA177DE1

##### Distribution

Atlanto-Mediterranean

##### Notes

Biological data: Univoltine. Flight period: IX-X. First record in Murcia Region.

#### Ematheudes punctellus

(Treitschke, 1833)

37480A67-6B70-5298-8FAB-FD6749D6665E

##### Distribution

Mediterranean-Asiatic

##### Notes

References: Derra and Hacker (1982). Biological data: Bivoltine. Flight period: V-X.

#### Polyochodes stipella

Chrétien, 1911

BB1A3E32-4C4E-5BF1-B827-6123A110CF23

##### Distribution

Atlanto-Mediterranean

##### Notes

Biological data: Univoltine. Flight period: VI. First record in Murcia Region.

#### Cryptoblabes gnidiella

(Millière, 1867)

B7CFDD97-DB5F-5284-84EF-17BD094BA727

##### Distribution

Mediterranean-Asiatic

##### Notes

References: Agenjo (1952). Biological data: Polyvoltine. Flight period: VII-VIII.

#### Pempeliella ardosiella

(Ragonot, 1887)

B8B718BA-BECE-52C0-9A33-3F0DB59E18A9

##### Distribution

Atlanto-Mediterranean

##### Notes

References: Slamka (2019). Biological data: Polyvoltine. Flight period: V-VII.

#### Huertasiella italogallicella

(Millière, 1883)

A8CD58EE-AEEC-5D4E-899F-590124D2AD11

##### Distribution

Atlanto-Mediterranean

##### Notes

Biological data: Bivoltine. Flight period: IX. First record in Murcia Region.

#### Uncinus hispanella

(Staudinger, 1859)

BA4E0A35-5AB3-5E78-9D23-2F6BEF8F163C

##### Distribution

Mediterranean-Asiatic

##### Notes

References: Slamka (2019). Biological data: Bivoltine. Flight period: V-VI.

#### Pseudosyria malacella

(Staudinger, 1870)

639A12E0-94E7-5DAE-AB2A-47F9D42E0C91

##### Distribution

Mediterranean-Asiatic

##### Notes

Biological data: Bivoltine. Flight period: II-VIII. First record in Murcia Region.

#### Pseudoinsalebria iberica

Slamka, Ylla & Macià, 2018

ED634723-CBA6-5519-80D2-3DFBC22A0BEC

##### Distribution

Endemic

##### Notes

References: Slamka et al. (2018), Slamka (2019). Biological data: Univoltine. Flight period: IV-V.

#### Asalebria florella

(Mann, 1862)

B8DB76BF-9753-509C-8D68-11DBBAB26348

##### Distribution

Eurasiatic

##### Notes

References: Zerny (1914). Biological data: Bivoltine. Flight period: V-VIII.

#### Asalebria ferruginella

(Zerny, 1914)

01EB8731-84CA-5E98-9785-220A7855C6B1

##### Distribution

Mediterranean-Asiatic

##### Notes

References: Schmidt (1934), Slamka (2019). Biological data: Univoltine. Flight period: V.

#### Psorosa dahliella

(Zerny, 1914)

DCFB5389-24DD-59BF-B94A-DDDB530F1AD0

##### Distribution

Mediterranean-Asiatic

##### Notes

References: Slamka (2010). Biological data: Bivoltine. Flight period: V.

#### Psorosa ferrugatella

Turati, 1924

45B87397-2A29-5DAE-BA94-DE6EE2B868ED

##### Distribution

Mediterranean-Asiatic

##### Notes

References: Girdley et al. (2020). Biological data: Polyvoltine. Flight period: IV-VI.

#### Psorosa mediterranella

Amsel, 1953

A23EF6F8-C1FF-585E-B163-8B570D4C8432

##### Distribution

Mediterranean-Asiatic

##### Notes

References: Zerny (1914), Slamka (2019). Biological data: Polyvoltine. Flight period: V-VIII, X.

#### Alophia combustella

(Herrich-Schäffer, 1855)

DEF67F81-160C-5154-B943-B1BABB95439E

##### Distribution

Mediterranean-Asiatic

##### Notes

Biological data: Bivoltine. Flight period: III-X. First record in Murcia Region.

#### Rhodophaea formosa

(Haworth, 1811)

EC48BB17-AF3D-5F45-88F1-56BD78046182

##### Distribution

Eurasiatic

##### Notes

Biological data: Bivoltine. Flight period: V. First record in Murcia Region.

#### Sciota elegiella

(Zerny, 1929)

8B0E1772-8621-5647-84F1-C97DD6E03FD2

##### Distribution

Atlanto-Mediterranean

##### Notes

Biological data: Polyvoltine. Flight period: V-IX. First record in Murcia Region.

#### Faveria dionysia

(Zeller, 1846)

03B91912-4290-5897-9D35-3D4D7218ED7A

##### Distribution

Tropical

##### Notes

References: Slamka (2019). Biological data: Polyvoltine. Flight period: VIII, X.

#### Melathrix coenulentella

(Zeller, 1846)

30CF38D9-89D7-59BF-AC87-37BFED1870B4

##### Distribution

Mediterranean-Asiatic

##### Notes

Biological data: Bivoltine. Flight period: II, VI, IX-X. First record in Murcia Region.

#### Amphithrix sublineatella

(Staudinger, 1859)

F5F78982-1E11-51BF-9A49-223B90362ACB

##### Distribution

Mediterranean-Asiatic

##### Notes

References: Caradja (1910), Agenjo (1952). Biological data: Bivoltine. Flight period: IV-X.

#### Oxybia transversella

(Duponchel, 1836)

DAE30773-A43D-5939-9BDB-A64D799C84A0

##### Distribution

Mediterranean-Asiatic

##### Notes

References: Derra and Hacker (1982), De Prins (1985). Biological data: Polyvoltine. Flight period: II-III, V-X.

#### Denticera divisella

(Duponchel, 1843)

444EA283-B578-5C1A-A3B1-677052C800A6

##### Distribution

Tropical

##### Notes

References: De Prins (1985). Biological data: Bivoltine. Flight period: II, VIII-XI.

#### Oncocera semirubella

(Scopoli, 1763)

4D024112-5A2C-53E6-BA85-CDD8EB70D86E

##### Distribution

Palaearctic

##### Notes

Biological data: Polyvoltine. Flight period: V, VIII-IX. First record in Murcia Region.

#### Etiella zinckenella

(Treitschke, 1832)

97D1F5DB-69DE-5CFA-AACE-A87B61EFB115

##### Distribution

Cosmopolitan

##### Notes

References: Agenjo (1948), Derra and Hacker (1982). Biological data: Polyvoltine. Flight period: V-IX.

#### Pima boisduvaliella

(Guenée, 1845)

57B71507-332C-544C-89AC-F48FCFA032A2

##### Distribution

Holarctic

##### Notes

Biological data: Bivoltine. Flight period: II-V. First record in Murcia Region.

#### Merulempista turturella

(Zeller, 1848)

9F7C46A4-B250-562D-9E42-F3DEA4D2A74E

##### Distribution

Mediterranean-Asiatic

##### Notes

References: Asselbergs (1993). Biological data: Bivoltine. Flight period: IV-V, VII-IX.

#### Merulempista azrouella

(D. Lucas, 1933)

BAB965E5-0DD5-5061-AD01-F7D13ACC4A94

##### Distribution

Atlanto-Mediterranean

##### Notes

Biological data: Bivoltine. Flight period: II-IV, VII. First record in Murcia Region.

#### Tephris ochreella

Ragonot, 1893

C2EC597B-E2DC-5140-BAB6-251FCA1C3F0B

##### Distribution

Mediterranean-Asiatic

##### Notes

References: Vives-Moreno (1992), Vives-Moreno (2014), Slamka (2019). Biological data: Univoltine. Flight period: V.

#### Pempelia palumbella

(Denis & Schiffermüller, 1775)

FAAFAC44-3054-5AAA-998D-B5B3322C2AFE

##### Distribution

Eurasiatic

##### Notes

References: Slamka (2019). Biological data: Bivoltine. Flight period: I-XII.

#### Pempelia albariella

Zeller, 1839

D6B7DA59-09FC-52EE-9D21-C75CF6CD79CC

##### Distribution

Mediterranean-Asiatic

##### Notes

Biological data: Bivoltine. Flight period: V-VII. First record in Murcia Region.

#### Pempelia genistella

(Duponchel, 1836)

621D0868-B6DD-5885-8399-CF0F5D50CD70

##### Distribution

Atlanto-Mediterranean

##### Notes

Biological data: Univoltine. Flight period: VIII. First record in Murcia Region.

#### Pempelia brephiella

(Staudinger, 1879)

37157B27-C710-5D2C-917E-193FCB97E507

##### Distribution

Mediterranean-Asiatic

##### Notes

References: Pérez de-Gregorio and Requena (2014), Slamka (2019). Biological data: Bivoltine. Flight period: II-VI, IX-XII.

#### Pempelia compositella

(Treitschke, 1835)

7DD749AF-7E8E-53CF-820D-3CA0C426D770

##### Distribution

Eurasiatic

##### Notes

Biological data: Bivoltine. Flight period: III-VIII. First record in Murcia Region.

#### Phycita roborella

(Denis & Schiffermüller, 1775)

071A0E06-D24C-539E-BCDA-01FF970A46D2

##### Distribution

Eurasiatic

##### Notes

Biological data: Univoltine. Flight period: IX. First record in Murcia Region.

#### Phycita diaphana

(Staudinger, 1870)

8356E7C7-E831-5565-8639-BE4CAF14E960

##### Distribution

Tropical

##### Notes

Biological data: Polyvoltine. Flight period: IV, IX-X. First record in Murcia Region.

#### Ceutholopha isidis

(Zeller, 1867)

553B882A-068F-533E-A049-5B974C370973

##### Distribution

Tropical

##### Notes

References: Girdley et al. (2018). Biological data: Polyvoltine. Flight period: V, VIII.

#### Dioryctria abietella

(Denis & Schiffermüller, 1775)

121A236C-9DA7-5673-8D78-F2914C7DA382

##### Distribution

Eurasiatic

##### Notes

Biological data: Bivoltine. Flight period: V, IX. First record in Murcia Region.

#### Dioryctria sylvestrella

(Ratzeburg, 1840)

E8274CFF-3F96-57EC-B048-73715FB8B2C3

##### Distribution

Eurasiatic

##### Notes

References: Zerny (1914). Biological data: Univoltine. Flight period: IX-X.

#### Dioryctria mendacella

(Staudinger, 1859)

EAB0DF15-3E87-5BC2-B24A-DAC1AF8FC30C

##### Distribution

Mediterranean-Asiatic

##### Notes

References: Knölke (2007). Biological data: Bivoltine. Flight period: II-X.

#### Dioryctria pineae

(Staudinger, 1859)

FD038B84-20BF-5677-9FE2-8AB88C2D383B

##### Distribution

Mediterranean-Asiatic

##### Notes

References: Pérez de-Gregorio and Requena (2014). Biological data: Univoltine. Flight period: IX-X.

#### Epischnia prodromella

(Hübner, 1799)

A6C3182F-AAEA-5857-AEBF-9A0B9EDDEDE3

##### Distribution

Mediterranean-Asiatic

##### Notes

References: Agenjo (1948). Biological data: Bivoltine. Flight period: IV-VII, IX, XII.

#### Epischnia illotella

Zeller, 1839

0665BCA0-0F7C-5054-B029-1D369B519051

##### Distribution

Mediterranean-Asiatic

##### Notes

References: Caradja (1910), Agenjo (1948), Agenjo (1952), Pérez de-Gregorio and Requena (2014). Biological data: Bivoltine. Flight period: II-XI.

#### Epischnia asteris

Staudinger, 1871

17ABCAFD-A12A-5E16-8FBD-80056A8584BF

##### Distribution

Atlanto-Mediterranean

##### Notes

References: Agenjo (1948), Pérez de-Gregorio and Requena (2014). Biological data: Bivoltine. Flight period: IV-VII, X.

#### Epischnia ampliatella

(Heinemann, 1864)

12381DC0-DDF0-5769-BE22-1EEBB0AE5BD3

##### Distribution

Eurasiatic

##### Notes

References: Girdley et al. (2020). Biological data: Univoltine. Flight period: II.

#### Nephopterix angustella

(Hübner, 1796)

22553273-51F0-5FD8-9AD4-1DEE10388E0D

##### Distribution

Eurasiatic

##### Notes

Biological data: Bivoltine. Flight period: VIII. First record in Murcia Region.

#### Acrobasis legatea

(Haworth, 1811)

C18D44B3-36B6-598D-B2D7-234C33E8651C

##### Distribution

Mediterranean-Asiatic

##### Notes

References: Derra and Hacker (1982). Biological data: Univoltine. Flight period: V-VIII.

#### Acrobasis bithynella

Zeller, 1848

6154404B-13EA-5737-AC83-82CF1BBAB1D7

##### Distribution

Mediterranean-Asiatic

##### Notes

Biological data: Univoltine. Flight period: VII-X. First record in Murcia Region.

#### Acrobasis obliqua

(Zeller, 1847)

1119A135-1FAF-585D-81EE-AC5596566C31

##### Distribution

Mediterranean-Asiatic

##### Notes

References: Caradja (1910). Biological data: Univoltine. Flight period: II-V.

#### Acrobasis romanella

(Millière, 1869)

D4119D31-DBBC-5118-94AF-6599758A5BB5

##### Distribution

Atlanto-Mediterranean

##### Notes

Biological data: Univoltine. Flight period: VIII-X. First record in Murcia Region.

#### Acrobasis sodalella

Zeller, 1848

E927AFD2-E62A-55BD-BB03-F0EB56E7FB4D

##### Distribution

Eurasiatic

##### Notes

Biological data: Univoltine. Flight period: VIII. First record in Murcia Region.

#### Acrobasis fallouella

(Ragonot, 1871)

7DC9A89A-9D8A-50CD-B26F-FF9D5785F071

##### Distribution

Mediterranean-Asiatic

##### Notes

Biological data: Univoltine. Flight period: VI-VIII. First record in Murcia Region.

#### Acrobasis centunculella

(Mann, 1859)

ED605580-82FA-5C63-9DAE-153ECBB7BCE6

##### Distribution

Mediterranean-Asiatic

##### Notes

References: Caradja (1910). Biological data: Univoltine. Flight period: II-X.

#### Acrobasis obtusella

(Hübner, 1796)

B2819911-F4FD-5AA1-B837-13ED1E1A67AD

##### Distribution

Eurasiatic

##### Notes

Biological data: Univoltine. Flight period: VI. First record in Murcia Region.

#### Apomyelois bistriatella

(Hulst, 1887)

C7D99C8C-09DB-5913-BBE3-C724F32F0BCF

##### Distribution

Holarctic

##### Notes

Biological data: Univoltine. Flight period: VIII. First record in Murcia Region.

#### Apomyelois ceratoniae

(Zeller, 1839)

6C783D22-AAB1-5DA4-8219-4EAD690FB458

##### Distribution

Cosmopolitan

##### Notes

Biological data: Polyvoltine. Flight period: IV-V, VII-X. First record in Murcia Region.

#### Eurhodope rosella

(Scopoli, 1763)

A2E0146B-9D8C-5F52-9DC1-660E6E94454D

##### Distribution

Eurasiatic

##### Notes

Biological data: Univoltine. Flight period: VI-VII. First record in Murcia Region.

#### Eurhodope cruentella

(Duponchel, 1843)

3AFE583D-0F31-5685-9125-FC0897C3BD1A

##### Distribution

Mediterranean-Asiatic

##### Notes

References: Caradja (1916), Pérez de-Gregorio and Requena (2008c). Biological data: Univoltine. Flight period: IV-VI.

#### Myelois circumvoluta

(Geoffroy in Fourcroy, 1785)

FCF630A5-56BF-544C-93AE-ED53D6A90766

##### Distribution

Eurasiatic

##### Notes

Biological data: Univoltine. Flight period: IV. First record in Murcia Region.

#### Myelois fuscicostella

Mann, 1861

C6C2217F-E663-5DF3-A76D-060B33D8E2BD

##### Distribution

Mediterranean-Asiatic

##### Notes

Biological data: Univoltine. Flight period: III-V. First record in Murcia Region.

#### Valdovecaria hispanicella

(Herrich-Schäffer, 1855)

E8828C6A-2B62-522E-B6E4-F1F80046BE6B

##### Distribution

Atlanto-Mediterranean

##### Notes

References: Pérez de-Gregorio and Requena (2010). Biological data: Univoltine. Flight period: IV-IX.

#### Pterothrixidia rufella

(Duponchel, 1836)

B1F701B8-7A72-5A82-ABFE-F91976981FD6

##### Distribution

Mediterranean-Asiatic

##### Notes

References: Caradja (1910). Biological data: Univoltine. Flight period: VI-VIII.

#### Seeboldia korgosella

Ragonot, 1887

899F4A61-55B0-5E9A-8B01-1BA709B82D7E

##### Distribution

Eurasiatic

##### Notes

Biological data: Univoltine. Flight period: IV, VIII. First record in Murcia Region.

#### Epischidia fulvostrigella

(Eversmann, 1844)

42F6CA84-5A08-5643-AA28-995718573BD4

##### Distribution

Mediterranean-Asiatic

##### Notes

Biological data: Univoltine. Flight period: VIII-IX. First record in Murcia Region.

#### Gymnancyla ruscinonella

(Ragonot, 1888)

E1969B6F-A5BB-51D8-8298-B892D50F05AE

##### Distribution

Atlanto-Mediterranean

##### Notes

References: Gastón and Vives (2018). Biological data: Bivoltine. Flight period: III-VI, VIII-IX.

#### Gymnancyla hillneriella

Gastón & Vives, 2018

2C69656B-1D31-590A-88A9-BEABA63AF15F

##### Distribution

Endemic

##### Notes

References: Gastón and Vives (2018). Biological data: Bivoltine. Flight period: II-IX.

#### Gymnancyla canella

(Denis & Schiffermüller, 1775)

0C044E3F-1B8E-5ACC-BEAD-C447BADDF7E8

##### Distribution

Mediterranean-Asiatic

##### Notes

References: Gastón and Vives (2018). Biological data: Univoltine. Flight period: VIII.

#### Metallostichodes nygrocianella

(Constant, 1865)

C38D623B-B870-500B-9BD0-0F472571C307

##### Distribution

Mediterranean-Asiatic

##### Notes

Biological data: Bivoltine. Flight period: IX. First record in Murcia Region.

#### Assara conicolella

(Constant, 1884)

2FA61D57-6DC3-559A-9B3E-7E96FE849C80

##### Distribution

Mediterranean-Asiatic

##### Notes

References: Zerny (1914). Biological data: Univoltine. Flight period: VIII.

#### Euzophera pinguis

(Haworth, 1811)

AC1E1019-CD19-5DF7-B299-A1DD0B34908C

##### Distribution

Eurasiatic

##### Notes

Biological data: Bivoltine. Flight period: V, VII-X. First record in Murcia Region.

#### Euzophera lunulella

(O. Costa, 1836)

49E66EA3-8D08-5F6D-87B7-30CF57572EF2

##### Distribution

Mediterranean-Asiatic

##### Notes

References: Zerny (1914), Derra and Hacker (1982), Pérez de-Gregorio and Requena (2008b), Palacios and Abad (2010). Biological data: Univoltine. Flight period: VI-IX.

#### Euzophera osseatella

(Treitschke, 1832)

612758CD-8ED3-5E95-93F1-F3AC38D005FA

##### Distribution

Mediterranean-Asiatic

##### Notes

Biological data: Polyvoltine. Flight period: V, X. First record in Murcia Region.

#### Euzopherodes vapidella

(Mann, 1857)

9435B0DE-F5F2-5346-84D3-E6AA1CA64A9D

##### Distribution

Mediterranean-Asiatic

##### Notes

Biological data: Polyvoltine. Flight period: II-V, X-XI. First record in Murcia Region.

#### Nyctegretis ruminella

(La Harpe, 1860)

D08B34FF-622C-5841-804B-0A5FC857910E

##### Distribution

Mediterranean-Asiatic

##### Notes

References: Pérez de-Gregorio and Requena (2014). Biological data: Bivoltine. Flight period: V-VI, VIII.

#### Ancylosis cinnamomella

(Duponchel, 1836)

5E998E23-CC38-54DE-851A-F71CCEE43C11

##### Distribution

Eurasiatic

##### Notes

References: De Prins (1985). Biological data: Bivoltine. Flight period: I-X.

#### Ancylosis uncinatella

(Ragonot, 1890)

B237244F-7F60-504F-9B95-6B017E2AFC32

##### Distribution

Atlanto-Mediterranean

##### Notes

References: Agenjo (1962), Derra and Hacker (1982). Biological data: Bivoltine. Flight period: III, V, VII.

#### Ancylosis maculifera

Staudinger, 1870

223DFB8E-A4CD-5880-93E0-F0E23A250BE3

##### Distribution

Eurasiatic

##### Notes

Biological data: Bivoltine. Flight period: V. First record in Murcia Region.

#### Ancylosis samaritanella

(Zeller, 1867)

F1FCC22E-0E6F-599A-9BD4-79F9FEFD817D

##### Distribution

Mediterranean-Asiatic

##### Notes

Biological data: Bivoltine. Flight period: VII. First record in Murcia Region.

#### Ancylosis roscidella

(Eversmann, 1844)

451A1832-1CE3-5FB3-A624-8162F6297028

##### Distribution

Eurasiatic

##### Notes

References: Roesler (1973). Biological data: Bivoltine.

#### Ancylosis gracilella

(Ragonot, 1887)

AE09AE31-4E07-53C9-9729-8A94853A5C7D

##### Distribution

Mediterranean-Asiatic

##### Notes

Biological data: Polyvoltine. Flight period: IV-V, IX. First record in Murcia Region.

#### Ancylosis harmoniella

(Ragonot, 1887)

A52C6515-2E0B-593D-AB41-DFC9E2F95847

##### Distribution

Mediterranean-Asiatic

##### Notes

Biological data: Bivoltine. Flight period: IV-V, X. First record in Murcia Region.

#### Ancylosis rhodochrella

(Herrich-Schäffer, 1852)

65D5628B-8ED5-57F7-8A4A-6CD3C00CE394

##### Distribution

Mediterranean-Asiatic

##### Notes

Biological data: Bivoltine. Flight period: V, VIII. First record in Murcia Region.

#### Ancylosis oblitella

(Zeller, 1848)

E525C474-85BE-598E-9B64-B6039E2CA510

##### Distribution

Eurasiatic

##### Notes

Biological data: Bivoltine. Flight period: IV, VII-XI. First record in Murcia Region.

#### Ancylosis calcariella

Ragonot, 1901

BC9CF625-1B14-5D15-9AAE-A02C058D4952

##### Distribution

Atlanto-Mediterranean

##### Notes

Biological data: Bivoltine. Flight period: IV-V, VII-X. First record in Murcia Region.

#### Ancylosis yerburii

(Butler, 1884)

83E72189-63C0-597B-BBEB-8688189CED0D

##### Distribution

Mediterranean-Asiatic

##### Notes

References: Bidzilya et al. (2020). Biological data: Bivoltine. Flight period: III-VII, IX.

#### Homoeosoma sinuella

(Fabricius, 1794)

66B75B88-CF88-5CA9-86F4-FF96DEB567C1

##### Distribution

Eurasiatic

##### Notes

References: Caradja (1910), Derra and Hacker (1982). Biological data: Bivoltine. Flight period: V-VIII.

#### Homoeosoma nebulella

(Denis & Schiffermüller, 1775)

08652440-6142-5989-B1BB-E725FA0FC88C

##### Distribution

Eurasiatic

##### Notes

References: Caradja (1910). Biological data: Polyvoltine. Flight period: VIII.

#### Homoeosoma nimbella

(Duponchel, 1837)

401CA697-754D-5C6C-B4D2-5613D03BFF2F

##### Distribution

Eurasiatic

##### Notes

References: Agenjo (1952). Biological data: Bivoltine. Flight period: V.

#### Phycitodes arenicola

(Chrétien, 1911)

CCADBE18-5A66-5639-BC06-103F21114234

##### Distribution

Atlanto-Mediterranean

##### Notes

Biological data: Univoltine. Flight period: V. First record in Murcia Region.

#### Phycitodes binaevella

(Hübner, 1813)

F19A08EC-DD99-530A-9386-9593213B9CFC

##### Distribution

Eurasiatic

##### Notes

Biological data: Univoltine. Flight period: III-V. First record in Murcia Region.

#### Phycitodes saxicola

(Vaughan, 1870)

3D9DC5D5-3029-524E-908A-8D144AD85B78

##### Distribution

Eurasiatic

##### Notes

References: Roesler (1973). Biological data: Bivoltine. Flight period: II-VI, VIII-XI.

#### Phycitodes lacteella

(Rothschild, 1915)

21DAB015-1641-5DFC-BABD-DAB70E2865EA

##### Distribution

Eurasiatic

##### Notes

Biological data: Univoltine. Flight period: II, VI, IX-X. First record in Murcia Region.

#### Phycitodes bentinckella

(Pierce, 1937)

835D6C61-9107-5B04-8EC4-F21F370B2F38

##### Distribution

Atlanto-Mediterranean

##### Notes

References: Roesler (1973). Biological data: Univoltine.

#### Phycitodes albatella

(Ragonot, 1887)

9A62225D-E44F-507C-929C-DBCE17D4E424

##### Distribution

Holarctic

##### Notes

References: Roesler (1973). Biological data: Polyvoltine. Flight period: VI.

#### Phycitodes inquinatella

(Ragonot, 1887)

18579E9D-E272-57FD-8EA4-C1A6AF7E7E44

##### Distribution

Mediterranean-Asiatic

##### Notes

Biological data: Bivoltine. Flight period: V, VII-VIII. First record in Murcia Region.

#### Archiephestia adpiscinella

(Chrétien, 1911)

F53CC5F6-082D-5C8D-B4AD-CF41FB2127AC

##### Distribution

Mediterranean-Asiatic

##### Notes

References: Leraut (2014). Biological data: Univoltine. Flight period: V-X.

#### Plodia interpunctella

(Hübner, 1813)

93C34B57-5F2E-56B0-BE47-0D17525A711F

##### Distribution

Cosmopolitan

##### Notes

Biological data: Polyvoltine. Flight period: V-IX. First record in Murcia Region.

#### Ephestia disparella

Ragonot, 1901

17EDE3B5-6E48-554A-A6AB-E6008D26B734

##### Distribution

Mediterranean-Asiatic

##### Notes

References: Roesler (1973). Biological data: Univoltine. Flight period: VI.

#### Ephestia parasitella

Staudinger, 1859

10B29580-86BE-5922-81B3-A0869A9A23AD

##### Distribution

Atlanto-Mediterranean

##### Notes

Biological data: Polyvoltine. Flight period: V-VI, IX. First record in Murcia Region.

#### Ephestia woodiella

Richards & Thomson, 1832

782FE72B-F955-52CD-AE43-FE2B3CD0A86F

##### Distribution

Eurasiatic

##### Notes

Biological data: Polyvoltine. Flight period: IX. First record in Murcia Region.

#### Ephestia kuehniella

(Zeller, 1879)

2E44A65E-ADA2-5BC7-ACA8-AB7E74B05679

##### Distribution

Cosmopolitan

##### Notes

Biological data: Polyvoltine. Flight period: VI, XII. First record in Murcia Region.

#### Ephestia welseriella

(Zeller, 1848)

06C726D2-5E4D-543E-8B87-CCE1A805690D

##### Distribution

Mediterranean-Asiatic

##### Notes

References: Zerny (1914), Derra and Hacker (1982). Biological data: Bivoltine. Flight period: V-VIII.

#### Cadra furcatella

(Herrich-Schäffer, 1851)

54C0C01A-9F13-5843-8ECF-EEAB62485D8C

##### Distribution

Mediterranean-Asiatic

##### Notes

Biological data: Bivoltine. Flight period: VI. First record in Murcia Region.

#### Cadra figulilella

(Gregson, 1871)

7FB071BC-ACBC-597D-A028-3C86FCA41171

##### Distribution

Cosmopolitan

##### Notes

Biological data: Polyvoltine. Flight period: V-X. First record in Murcia Region.

#### Cadra cautella

(Walker, 1863)

EC08A98A-E792-5427-A1FD-F9D8D71178D6

##### Distribution

Cosmopolitan

##### Notes

Biological data: Polyvoltine. Flight period: VIII. First record in Murcia Region.

#### Cadra calidella

(Guenée, 1845)

0F2C5CF0-B754-54C4-AB24-75DE4ABD3E0A

##### Distribution

Mediterranean-Asiatic

##### Notes

Biological data: Polyvoltine. Flight period: IX. First record in Murcia Region.

#### Pyralinae

59A5EF68-B6C1-5580-B840-64EDCE2CA745

#### Endotricha flammealis

(Denis & Schiffermüller, 1775)

5C010146-13D2-54D8-95D0-5C48F8B4B3FE

##### Distribution

Eurasiatic

##### Notes

Biological data: Bivoltine. Flight period: VI-IX. First record in Murcia Region.

#### Hypotia corticalis

(Denis & Schiffermüller, 1775)

1D92F0A5-F734-516D-92A8-F38EB84909BD

##### Distribution

Mediterranean-Asiatic

##### Notes

References: Agenjo (1952). Biological data: Bivoltine. Flight period: V-VIII.

#### Hypotia infulalis

Lederer, 1858

B7152D2B-36AE-5CDD-82EB-381448CAE0F2

##### Distribution

Mediterranean-Asiatic

##### Notes

References: Vives-Moreno (1992). Biological data: Bivoltine. Flight period: II-X.

#### Hypotia pectinalis

(Herrich-Schäffer, 1838)

526F0322-E337-54A1-936E-9A1832A29231

##### Distribution

Mediterranean-Asiatic

##### Notes

Biological data: Bivoltine. Flight period: IV-V, VII-VIII. First record in Murcia Region.

#### Hypotia miegi

(Ragonot, 1895)

7BAEEF1D-E0E5-5EDF-BE07-1DC0A51B4751

##### Distribution

Endemic

##### Notes

References: Ragonot (1895), Agenjo (1962), Slamka (2006). Biological data: Bivoltine. Flight period: II-III, V-IX.

#### Hypotia leucographalis

(Hampson, 1900)

188E575D-F81C-5C06-9531-3FFA7009B628

##### Distribution

Endemic

##### Notes

References: Hampson (1900), Agenjo (1952), Slamka (2006). Biological data: Bivoltine. Flight period: IV-V, VII-X.

#### Synaphe moldavica

(Esper, 1794)

1A268E1F-3F16-5DCF-B825-32DA85C1F013

##### Distribution

Mediterranean-Asiatic

##### Notes

Biological data: Univoltine. Flight period: V-VI. First record in Murcia Region.

#### Synaphe diffidalis

(Guenée, 1854)

722AE73C-61C0-5E1A-BE7C-2DF11AA2829A

##### Distribution

Atlanto-Mediterranean

##### Notes

References: Caradja (1916). Biological data: Univoltine. Flight period: II-V.

#### Synaphe predotalis

(Zerny, 1927)

1C57E773-BE72-5AEF-9449-29329762EB88

##### Distribution

Atlanto-Mediterranean

##### Notes

Biological data: Univoltine. Flight period: VI-VIII. First record in Murcia Region.

#### Synaphe punctalis

(Fabricius, 1775)

943D861D-FFCD-5B5A-A556-E285BB799642

##### Distribution

Eurasiatic

##### Notes

References: Slamka (2006). Biological data: Univoltine. Flight period: VII-VIII.

#### Pyralis farinalis

(Linnaeus, 1758)

D0AE5B16-E21E-5621-9F72-0FE6A2F0C16E

##### Distribution

Cosmopolitan

##### Notes

Biological data: Polyvoltine. Flight period: III-IV, VI-XI. First record in Murcia Region.

#### Aglossa pinguinalis

(Linnaeus, 1758)

AF4C1B6F-0186-5536-B584-3453255CC7E0

##### Distribution

Cosmopolitan

##### Notes

Biological data: Univoltine. Flight period: IV, IX-X. First record in Murcia Region.

#### Aglossa caprealis

(Hübner, 1809)

EED07796-5AC4-5E5B-87AD-06504E2800FE

##### Distribution

Cosmopolitan

##### Notes

Biological data: Univoltine. Flight period: VI. First record in Murcia Region.

#### Aglossa brabanti

Ragonot, 1884

6E7BDC6B-1D6A-5E92-8793-8D9F124379D2

##### Distribution

Atlanto-Mediterranean

##### Notes

References: Zerny (1914), Slamka (2006), Pérez de-Gregorio and Requena (2008a). Biological data: Univoltine. Flight period: V-VIII.

#### Aglosa mayrae

Ylla, Šumpich, Gastón, Huertas & Macià, 2017

7F3D22C5-799C-5056-BB12-FDABC58D4AAB

##### Distribution

Endemic

##### Notes

Biological data: Bivoltine. Flight period: IV-V. First record in Murcia Region.

#### Stemmatophora combustalis

(Fisher von Röslerstamm, 1842)

05EA0707-93D0-5356-B4FE-0761625F4A65

##### Distribution

Mediterranean-Asiatic

##### Notes

References: Derra and Hacker (1982). Biological data: Univoltine. Flight period: V-VIII.

#### Stemmatophora gadesalis

Ragonot, 1882

F1455122-10BF-533E-ABDB-3FC8AFE2E856

##### Distribution

Atlanto-Mediterranean

##### Notes

References: Caradja (1916). Biological data: Univoltine.

#### Stemmatophora vulpecalis

Ragonot, 1891

8FCB2901-4EC8-5DF6-B908-7BD6729F5CDE

##### Distribution

Atlanto-Mediterranean

##### Notes

References: Zerny (1914), Slamka (2006). Biological data: Univoltine. Flight period: VI-VIII.

#### Stemmatophora syriacalis

(Ragonot, 1895)

D88A5953-3505-572C-A7CF-06917243F870

##### Distribution

Mediterranean-Asiatic

##### Notes

Biological data: Univoltine. Flight period: VI-VIII. First record in Murcia Region.

#### Stemmatophora rungsi

(Leraut, 2000)

9F32FB2F-4E60-519E-9E8E-A63A7E6FB9C1

##### Distribution

Atlanto-Mediterranean

##### Notes

Biological data: Univoltine. Flight period: IX. First record in Murcia Region.

#### Stemmatophora brunnealis

(Treitschke, 1829)

45609B3D-DD3D-5DEF-82E1-84B62A4FAAE6

##### Distribution

Mediterranean-Asiatic

##### Notes

Biological data: Univoltine. Flight period: VIII-X. First record in Murcia Region.

#### Stemmatophora borgialis

(Duponchel, 1833)

88F2FAE2-BB33-5D57-86F0-D285135C4095

##### Distribution

Atlanto-Mediterranean

##### Notes

Biological data: Univoltine. Flight period: VII-X. First record in Murcia Region.

#### Maradana fuscolimbalis

(Ragonot, 1887)

14B8F838-54EA-5C38-95C2-416D1650A393

##### Distribution

Atlanto-Mediterranean

##### Notes

Biological data: Polyvoltine. Flight period: IV-XI. First record in Murcia Region.

#### Bostra obsoletalis

(Mann, 1884)

A01BE3E4-1D6E-56C4-8E5F-4E6E50A47885

##### Distribution

Mediterranean-Asiatic

##### Notes

References: De Prins (1985). Biological data: Bivoltine. Flight period: IV-IX.

#### Loryma egregialis

(Herrich-Schäffer, 1838)

BD40F19E-7A3C-5C7A-AAEE-158609647A9C

##### Distribution

Mediterranean-Asiatic

##### Notes

References: Zerny (1914), Agenjo (1952), Slamka (2006). Biological data: Bivoltine. Flight period: IV-X.

#### Hypsopygia costalis

(Fabricius, 1775)

249A0195-DFBA-54D0-ADA5-5BE4B614308C

##### Distribution

Holarctic

##### Notes

Biological data: Bivoltine. Flight period: VI-X. First record in Murcia Region.

#### Hypsopygia incarnatalis

(Zeller, 1847)

3A541C97-25A4-5D39-BBF9-F4A55BE7BBF8

##### Distribution

Mediterranean-Asiatic

##### Notes

Biological data: Univoltine. Flight period: IX-X. First record in Murcia Region.

## Analysis

The list includes 142 species in 67 genera and three subfamilies: Galleriinae (8 species), Phycitinae (107 species) and Pyralinae (27 species). Seventy-three new records (51%) from the Murcia Region are added to its Lepidopteran fauna.

The most species-rich subfamily, Phycitinae, comprises 77.6% of all genera and 75.3% of all species, while Pyralinae comprise 14.9% and 19.1% and Galleriinae with 7.5% and 5.6%, respectively (Table 1).

The European family of Pyralidae consists of 470 species (Leraut 2014), whilst the Iberian Pyralidae fauna comprise 262 extant species (Vives-Moreno 2014). Thus, to date, the number of species known from the Murcia Region accounts for approximately 30% of the European total and 54.1% of the Iberian species.

Alpha diversity indices applied to abundance data (2683 individuals of 136 species collected) showed a low dominance value of 0.96 (all taxa are equally present) and a Chao1 estimate of total species richness amongst 140 species (lower value) to 165.5 species (upper value) which is close to 142 species studied and foresees the addition of new species in the future.

Known Pyralidae diversity in the Murcia Region seem relatively rich when compared to those in other Iberian Regions and with the whole of the Iberian Peninsula, as for instance, similar Iberian Regions extensively surveyed like Catalonia (172 species; Dantart 2020) and Aragon (163 species; Redondo et al. 2017). This may be because intensive surveys have started only recently or because the biodiversity is greater closer to the temperate areas. However, we are sure that an increase in the sampling effort will allow adding new species to the of Pyralidae checklist from the Murcia Region.

The most species-rich Pyralidae genera in the Murcia Region are *Ancylosis* (11 species, 7.7%), *Acrobasis* (8 species, 5.6%), *Phycitoides* and *Stemmatophora* (*7* species, 4.9% each, respectively), *Pempelia, Ephestia* and *Hypotia* (5 species, 3.5% each, respectively) and *Aphomia, Cadra, Dioryctria, Epischinia, Aglossa* and *Synaphe* (4 species, 2.8% each, respectively). The majority of genera (12) are species-poor (2-3 species) or known in the Murcia Region from a single species (42 genera).

Species richness varies substantially amongst the different bioclimatic areas of the Murcia Region (Fig. 1). The Thermomediterranean area has the most diverse Pyralidae fauna with 116 species recorded, followed by the Mesomediterranean area with 73 species, while the Supra- and Oromediteranean areas appear to be less diverse with 39 species (Table 2). In each of these areas, 54 species are unique in the Thermo-, 10 in Meso- and four in Supra- and Oromediterranean areas, while 41 species were recorded in two areas and 26 in the three studied areas. Approximately 47.8% of the species can be considered specialists in a given bioclimatic area, while the other 52.2% can be considered as opportunists of different types of vegetation that characterise each of the bioclimatic areas. The detailed data for the bioclimatic areas of Pyralidae in the Murcia Region are summarised in Table 2.

Chorological analysis for the family Pyralidae in the Region of Murcia showed that the Mediterranean chorotype is the most abundant with 59.2% of the total, which is consistent with the geographical position of the study area. Amongst these, the Asiatic-Mediterranean elements (41.5%) are more frequent than the Atlanto-Mediterranean elements (17.6%). On the other hand, the elements of wide distribution, such as the Eurasiatic, Holarctic and Palaearctic (24.6%), are the most common in the mountainous biotopes of the centre and north of the study area, while the tropical and cosmopolitan species (12.0%) have their origin mainly in Africa. The presence of opportunistic species is due to the agricultural crop fields that dominate part of the Murcian territory. The Iberian endemisms are represented with six species (4.2%).

Regarding the biology of the species, the environmental conditions of the study area, which affect the availability of trophic resources for reproduction, suggest that most of the species are bivoltins (40.8%) and univoltins (38.0%), while the rest are polyvoltins (21.1%). Most of the Phyctinae recorded species feed on plant species belonging to the Asteraceae, Cistaceae, Fabaceae, Pinaceae, Fagaceae, Oleaceae, Chenopodiaceae and Lamiaceae families, amongst others. The most particular cases are those related to the genera *Cadra*, *Ephestia* and *Plodia* which are pests on stored products. Some species, such as *Apomyeolisceratoniae* (Zeller), *Cryptoblabesgnidiella* (Millière), *Etiellazinckeniella* (Treitschke) and *Euzopherapinguis* (Haworth) must be controlled since they are agricultural crop pests. Many of the species of the subfamily Galleriinae live in bee, bumblebee or wasp nests as well as on plant detritus. Others, such as *Aphomiasabella* (Hampson) and *Cathayiainsularum* (Speidel & Schmitz), are parasites of palm trees (*Phoenix* spp.). In relation to the subfamily Pyralinae, most of the species feed on plant and animal detritus with *Pyralisfarinalis* (Linnaeus) being also a particular pest on cereal flour. Finally, the food source and diet of 27.4% of species are unknown, so it will be necessary to carry out complementary studies for further biological understanding.

Some taxa cited in the references have been removed from the checklist as *Epischniamuscidella* Ragonot, cited in Caradja (1916), because it is distributed in Turkey (Leraut 2014); *Hypotiasyrtalis* (Ragonot) also cited in Caradja (1916) was removed in Slamka (2006) and Vives-Moreno (2014); *Psorosanucleolella* (Möschler) cited in Zerny (1914) from Sierra Espuña mountains was removed according to Slamka (2019); *Tephriscyriella* (Erschoff), cited from Sierra Espuña mountains in Vives-Moreno (1992) and later corrected as *T.ochreella* Ragonot in Vives-Moreno (2014) and Slamka (2019); *Laristaniaalbipunctella* (Chrétien), cited in Palm (2012), Leraut (2014) and Vives-Moreno (2014), has been cited as a new species *Pseudoinsalebriaiberica* Slamka, Ylla & Macià (Slamka et al. 2018). Finally, *Gymnancylahornigii* (Lederer) was cited in Girdley et al. (2019), but misidentified with *Epischidiafulvostrigella* (Eversmann) according to morphological and barcode data (unpublished data).

## Discussion

Prior to our investigation, the number of known Pyralidae moth species in the Murcia Region was 69. Our study increases this number to a total of 142, based on an examination of museum specimens, published records and sampled individuals, accounting for 54.1% of all of the Iberian species known. This study presents an updated checklist of current Pyralidae moth species with their distribution and biological information for the Murcia Region in the south-eastern Iberian Peninsula.

This study serves as both a guide for collection in the poorly sampled south-western European continent and a comprehensive reference list with the Pyralidae taxa and localities where conservation is an important priority for policy-makers, conservation planners and for the management of insect diversity in Spain.

We encourage lepidopterists holding additional data on systematically collected pyralids to produce an updated dataset. Additionally, new intensive surveys in adjacent regions are being conducted and unknown specimens are continuously identified to species level.

## Figures and Tables

**Table 1.** Numbers and percentages of known genera and species recorded for each subfamily in Murcia Region.

**Subfamilies**	**Genus richness**	% **Genus**	**Species richness**	% **Species**
Galleriinae	5	7.5	8	5.6
Phycitinae	52	77.6	107	75.3
Pyralinae	10	14.9	27	19.1
**Total**	67	100	142	100

**Table 2.** List of unique species in each bioclimatic area or in more than one bioclimatic area.

Oro- and Suprameditarreneam	*Acrobasisobtusella* (Hübner, 1796)
	*Phycitaroborella* ([Denis & Schiffermüller], 1775)
	*Eurhodoperosella* (Scopoli, 1763)
	*Cadrafurcatella* (Herrich-Schäffer, 1851)
	
Mesomediterraneam	*Huertasiellaitalogallicella* (Millière, 1883)
	*Rhodophaeaformosa* (Haworth, 1811)
	*Pempeliaalbariella* Zeller, 1839
	*Pempeliagenistella* (Duponchel, 1836)
	*Seeboldiakorgosella* Ragonot, 1887
	*Ancylosisrhodochrella* (Herrich-Schäffer, 1852)
	*Homoeosomanebulella* ([Denis & Schiffermüller], 1775)
	*Phycitodesarenicola* (Chrétien, 1911)
	*Synaphepunctalis* (Fabricius, 1775)
	*Stemmatophorarungsi* (Leraut, 2000)
	
Thermomediterraneam	*Achroiagrisella* (Fabricius, 1794)
	*Aphomiamurciella* Zerny, 1914
	*Aphomiasabella* (Hampson, 1901)
	*Lamoriazelleri* (Joannis, 1932)
	*Peoriacremoricosta* (Ragonot, 1895)
	*Peoriatranslucidella* (Chrétien, 1911)
	*Polyochodesstipella* Chrétien, 1911
	*Cryptoblabesgnidiella* (Millière, 1867)
	*Pseudosyriamalacella* (Staudinger, 1870)
	*Psorosaferrugatella* Turati, 1924
	*Sciotaelegiella* (Zerny, [1929])
	*Faveriadionysia* (Zeller, 1846)
	*Melathrixcoenulentella* (Zeller, 1846)
	*Denticeradivisella* (Duponchel, [1843])
	*Merulempistaturturella* (Zeller, 1848)
	*Merulempistaazrouella* (D. Lucas, 1933)
	*Tephrisochreella* Ragonot, 1893
	*Phycitadiaphana* (Staudinger, 1870)
	*Ceutholophaisidis* (Zeller, 1867)
	*Epischniaampliatella* (Heinemann, 1864)
	*Nephopterixangustella* (Hübner, 1796)
	*Acrobasissodalella* Zeller, 1848
	*Apomyeloisbistriatella* (Hulst, 1887)
	*Myeloiscircumvolut*a (Geoffroy in Fourcroy, 1785)
	*Epischidiafulvostrigella* (Eversmann, 1844)
	*Gymnancylahillneriella* Gastón & Vives, 2018
	*Gymnancylacanella* ([Denis & Schiffermüller], 1775)
	*Metallostichodesnygrocianella* (Constant, 1865)
	*Assaraconicolella* (Constant, 1884)
	*Euzopheraosseatella* (Treitschke, 1832)
	*Euzopherodesvapidella* (Mann, 1857)
	*Nyctegretisruminella* (La Harpe, 1860)
	*Ancylosisuncinatella* (Ragonot, 1890)
	*Ancylosismaculifera* Staudinger, 1870
	*Ancylosissamaritanella* (Zeller, 1867)
	*Ancylosiscalcariella* Ragonot, 1901
	*Ancylosisyerburii* (Butler, 1884)
	*Phycitodesbinaevella* (Hübner, [1813])
	*Phycitodeslacteella* (Rothschild, 1915)
	*Phycitodesinquinatella* (Ragonot, 1887)
	*Plodiainterpunctella* (Hübner, [1813])
	*Ephestiadisparella* Ragonot, 1901
	*Ephestiawoodiella* Richards & Thomson, 1832
	*Ephestiakuehniella* (Zeller, 1879)
	*Cadracautella* (Walker, 1863)
	*Cadracalidella* (Guenée, 1845)
	*Hypotiainfulalis* Lederer, 1858
	*Hypotiapectinalis* (Herrich-Schäffer, 1838)
	*Hypotialeucographalis* (Hampson, 1900)
	*Pyralisfarinalis* (Linnaeus, 1758)
	*Aglossapinguinalis* (Linnaeus, 1758)
	*Aglossacaprealis* (Hübner, [1809])
	*Hypsopygiacostalis* (Fabricius, 1775)
	*Hypsopygiaincarnatalis* (Zeller, 1847)
	
Oro-, Supra- and Mesomediterranean	*Aphomiasociella* (Linnaeus, 1758)
	*Uncinushispanella* (Staudinger, 1859)
	*Acrobasisromanella* (Millière, 1869)
	*Pterothrixidiarufella* (Duponchel, 1836)
	*Synaphemoldavica* (Esper, 1794)
	
Meso- and Thermomediterranean	*Cathayiainsularum* (Speidel & Schmitz, 1991)
	*Pseudoinsalebriaiberica* Slamka, Ylla & Macià, 2018
	*Psorosamediterranella* Amsel, 1953
	*Oncocerasemirubella* (Scopoli, 1763)
	*Pimaboisduvaliella* (Guenée, 1845)
	*Pempeliabrephiella* (Staudinger, 1879)
	*Dioryctriasylvestrella* (Ratzeburg, 1840)
	*Dioryctriapineae* (Staudinger, 1859)
	*Apomyeloisceratoniae* (Zeller, 1839)
	*Eurhodopecruentella* (Duponchel, [1843])
	*Myeloisfuscicostella* Mann, 1861
	*Valdovecariahispanicella* (Herrich-Schäffer, 1855)
	*Gymnancylaruscinonella* (Ragonot, 1888)
	*Euzopherapinguis* (Haworth, 1811)
	*Ancylosisgracilella* (Ragonot, 1887)
	*Ancylosisharmoniella* (Ragonot, 1887)
	*Ancylosisoblitella* (Zeller, 1848)
	*Phycitodessaxicola* (Vaughan, 1870)
	*Archiephestiaadpiscinella* (Chrétien, 1911)
	*Ephestiaparasitella* Staudinger, 1859
	*Ephestiawelseriella* (Zeller, 1848)
	*Cadrafigulilella* (Gregson, 1871)
	*Hypotiacorticalis* ([Denis & Schiffermüller], 1775)
	*Hypotiamiegi* (Ragonot, 1895)
	*Synaphediffidalis* (Guenée, 1854)
	*Synaphepredotalis* (Zerny, 1927)
	*Aglossabrabanti* Ragonot, 1884
	*Aglosamayrae* Ylla, Šumpich, Gastón, Huertas & Macià, 2017
	*Stemmatophorasyriacalis* (Ragonot, 1895)
	*Stemmatophoraborgialis* (Duponchel, [1833])
	*Maradanafuscolimbalis* (Ragonot, 1887)
	*Lorymaegregialis* (Herrich-Schäffer, 1838)
	
Oro- and Supra- and Thermomediterranean	*Alophiacombustella* (Herrich-Schäffer, 1855)
	*Dioryctriaabietella* ([Denis & Schiffermüller], 1775)
	*Acrobasislegatea* (Haworth, 1811)
	*Phycitodesalbatella* (Ragonot, 1887)
	
All areas	*Galleriamellonella* (Linnaeus, 1758)
	*Lamoriaanella* ([Denis & Schiffermüller], 1775)
	*Ematheudespunctellus* (Treitschke, 1833)
	*Pempeliellaardosiella* (Ragonot, 1887)
	*Asalebriaflorella* (Mann, 1862)
	*Amphithrixsublineatella* (Staudinger, 1859)
	*Oxybiatransversella* (Duponchel, 1836)
	*Etiellazinckenella* (Treitschke, 1832)
	*Pempeliapalumbella* ([Denis & Schiffermüller], 1775)
	*Pempeliacompositella* (Treitschke, 1835)
	*Dioryctriamendacella* (Staudinger, 1859)
	*Epischniaprodromella* (Hübner, [1799])
	*Epischniaillotella* Zeller, 1839
	*Epischniaasteris S*taudinger, 1871
	*Acrobasisbithynella* Zeller, 1848
	*Acrobasisobliqua* (Zeller, 1847)
	*Acrobasisfallouella* (Ragonot, 1871)
	*Acrobasiscentunculella* (Mann, 1859)
	*Euzopheralunulella* (O. Costa, [1836])
	*Ancylosiscinnamomella* (Duponchel, 1836)
	*Homoeosomasinuella* (Fabricius, 1794)
	*Endotrichaflammealis* ([Denis & Schiffermüller], 1775)
	*Stemmatophoracombustalis* (Fisher von Röslerstamm, [1842])
	*Stemmatophoravulpecalis* Ragonot, 1891
	*Stemmatophorabrunnealis* (Treitschke, 1829)
	*Bostraobsoletalis* (Mann, 1884)
